# Identifying Complex Emotions in Alexithymia Affected Adolescents Using Machine Learning Techniques

**DOI:** 10.3390/diagnostics12123188

**Published:** 2022-12-16

**Authors:** Stephen Dass ArulDass, Prabhu Jayagopal

**Affiliations:** School of Information Technology and Engineering, Vellore Institute of Technology, Vellore 632014, India

**Keywords:** affective computing, brainwave signals, electroencephalogram (EEG), emotion classification, feature selection distant discriminant (FSDD), multimodal stimulation, support vector machine, uniform manifold approximation and projection (UMAP)

## Abstract

Many scientific researchers’ study focuses on enhancing automated systems to identify emotions and thus relies on brain signals. This study focuses on how brain wave signals can be used to classify many emotional states of humans. Electroencephalography (EEG)-based affective computing predominantly focuses on emotion classification based on facial expression, speech recognition, and text-based recognition through multimodality stimuli. The proposed work aims to implement a methodology to identify and codify discrete complex emotions such as pleasure and grief in a rare psychological disorder known as alexithymia. This type of disorder is highly elicited in unstable, fragile countries such as South Sudan, Lebanon, and Mauritius. These countries are continuously affected by civil wars and disaster and politically unstable, leading to a very poor economy and education system. This study focuses on an adolescent age group dataset by recording physiological data when emotion is exhibited in a multimodal virtual environment. We decocted time frequency analysis and amplitude time series correlates including frontal alpha symmetry using a complex Morlet wavelet. For data visualization, we used the UMAP technique to obtain a clear district view of emotions. We performed 5-fold cross validation along with 1 s window subjective classification on the dataset. We opted for traditional machine learning techniques to identify complex emotion labeling.

## 1. Introduction

**Figure 1:**
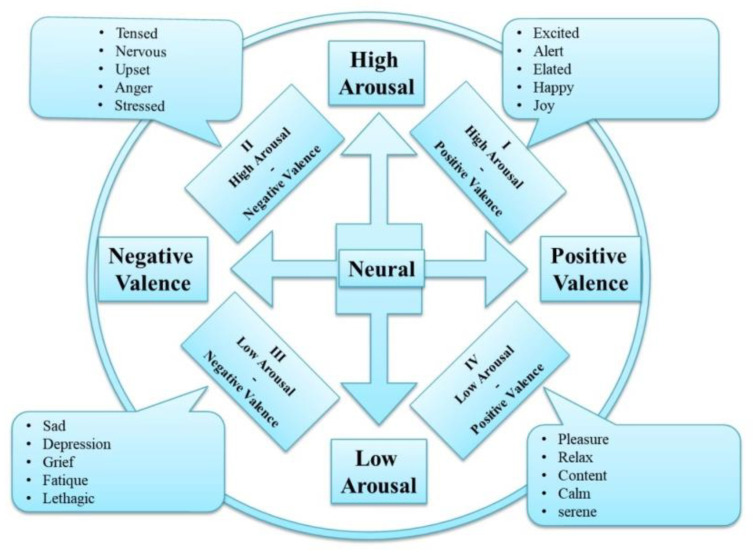
Circumplex emotional model to exhibit complex emotions.

Emotional interactions are advantageous for several applications relevant to health care since they have a considerable impact on cognitive functions of the human brain such as perception, memory, problem-solving, and learning. The main goal of affective computing is to program machines and experience emotion. It is also about teaching machines to recognize and differentiate between different human emotions and then to react appropriately. One of the most cutting-edge methods and designs for emotion analysis at the moment is the classification of emotions based on brain signals. The development of brain science has revealed that the brain creates human emotions. Numerous applications connected to brain waves and emotions result from this. Numerous research-minded individuals have proposed and used various approaches for categorizing brain wave emotions. EEG signals are predominantly used for real-time application. The main aim of this study is to identify complex discrete emotions (e.g., pleasure and grief) using machine learning techniques. EEG signals surpass temporal resolution for information processing. In recent years, research on emotion classification using EEG signals has often targeted two categories of emotion rooted in psychological models, basic emotional models known as circumplex valence and arousal [1], as shown in Figure 1, and later, in discrete models, basic emotions such as, anger, sadness, happiness, fear, surprise, and disgust.

We deploy the circumplex model, which we derived from Zhao et al.’s research [2], in which grief from a negative shade of emotion is extracted from EEG signals using a machine learning algorithm. According to an Oxford University Press publication, “One of the essential features of emotion is considered to be pleasure. It can be characterized as the affirmative assessment that serves as the foundation for many more elaborate assessments like “agreeable” or “pleasant”” [3]. One of the hottest study areas in emotion categorization was emotion recognition using deep learning algorithms. Convolution neural networks (CNNs) [4] in particular show promising results and outperform classical machine learning in identifying emotional states. Although there are numerous benefits to this, there are certain restrictions on EEG signals for brain–computer interface (BCI) [5] classification because of the limited training data that are currently available. To determine the difference between two emotional states, the feature-ranking method [6] is applied to the dataset to minimize its dimensionality and investigate its channel and frequency bands. Both emotions (pleasure and grief) should be analyzed because they have different electrophysiological correlations and generalizations to the FAA. In order to fully understand the efficiency of support vector machine (SVM) [7], we eventually turned to using Spearman’s correlation coefficient between subjective rating and subject-specific classification accuracy.

A person with alexithymia is unable to reveal or explain their emotional condition to others due to emotional blindness. In addition, they are unable to comprehend the emotions of others. The brain’s various cortices, including the anterior cingulate cortex, anterior insula, orbitofrontal cortex, medial temporal gyrus, and superior temporal sulcus, suffer from axing of gray matter, which is the root of alexithymia [8]. In addition, neuroscientists have researched to find the loop between alexithymia and the derivation of the two hemispheres and also deficiencies in the amygdala [9]. Adolescents can face issues in exhibiting their real emotions. Trying to exhibit a fake emotion leads to emotional immaturity. Most alexithymia affected adolescents have issues from their childhood practices and routinely later develop alexithymia or lack of emotional interest. This is due to continuous stressful and depression events happening in their life.

From 2011, Lebanon received more than one million Syrian refugees. The journey of evacuation is still continuing, creating a negative impact on social relationships and the economy and politics. These impacts lead to a lack of economic growth, and job scarcity leads to changes in human behavior such as highly aggressive nature and violence. Adolescents in Lebanon who experience ongoing negative effects deal with secondary health problems such as stress that cause mental illnesses such as sleeplessness, anxiety, alexithymia, and melancholy. The major goal of this study is to better understand these elements by relating the violent nature of Lebanon’s adolescents and alexithymia. This study’s implication and data description are borrows from the study of Sfeir et al. [10].

### 1.1. Objective and Problem Statement

This study’s key innovation is that we attempted to categorize complex affective states, which have only infrequently been investigated from EEG signals. We emphasize pleasure as an affiliative emotion that serves as the catalyst for prosocial behavior and the development of deep interpersonal relationships between numerous people as well as communal bonding, care, and wellbeing. In contrast, grief mimics a negative state of mental suffering associated with confusion, delusions, trouble relating to situations and people, paranoia, or hallucinations, and it focuses especially on the emotional response to loss based on physical, social, cultural, spiritual, cognitive, behavioral, and philosophical dimensions.

Appropriate stimulation of affective states veraciously and ethically in an analytical and practical environment is a critical challenge in emotion recognition research and, moreover, classifying adolescent emotion has added more complexity to research.

### 1.2. Related Works

Electroencephalography (EEG) data were used by Omid Bazgir et al. [11] to establish an emotion classification system based on the valence/arousal paradigm. By adopting the same dimensionality, the authors apply principle component analysis (PCA) to the retrieved features. Emotional states are defined using support vector machines (SVMs), K-nearest neighbors (KNNs), and artificial neural networks (ANNs). When used on the “DEAP” dataset, the recommended methodology fared better than pre-existing techniques.

Eleonora De Filippi et al. [12] used a strategy for distinguishing distinct complex emotions such as tenderness and anguish that can be gleaned from EEG. EEG-based affective computing uses varied proportions of emotion-based classification and is widely utilized in passive elicitation using single-modality stimuli. As a result, the authors combined emotional self-induction with a multimodal digital environment, incorporating both passive and active excerption tactics to record electrophysiological data throughout emotion-evoking trials. For the purpose of using complex Morlet wavelet convolution, the authors deduced correlational and time-frequency properties, including frontal-alpha asymmetry (FAA). Extensive research was carried out with outcomes of within-subject emotion categories using 1 s windows as an exemplar and trial-unique cross-validation. A support vector machine learning classifier with low operational complexity was implemented.

A paradigm for emotion recognition was put forth by Li et al. [13] to identify emotions. The authors implement binary gray wolf optimization (BGWO) to optimize the feature matrix and tunable Q-factor wavelet transform (TQWT) techniques to pre-process electroencephalogram (EEG) signals, then build the classifier utilizing a support vector machine (SVM). This study was conducted using a 32-subject DEAP-2012 dataset and a 6-fold cross-validation. The presented methodology was competent in discriminating emotion from physiological data with excellent specificity, sensitivity, and accuracy.

With the aid of various machine learning techniques such as support vector machine (SVM), K-nearest neighbor, linear discriminant analysis, logistic regression, and decision trees, Vikrant Doma and Matin Pirouz [14] compared all methodologies used to recognize emotion using brain signals measured using an electroencephalogram (EEG). Some health conditions, such as alexithymia, are examined with principal component analysis (PCA) to reduce dimensionality. EEG signals can demonstrate variations in electrical potential that result in neural communication networks that are researched for emotion analysis. The DEAP multimodal dataset was utilized by the authors to analyze human affective states. The outcome of the comparison research demonstrates that several classification models can be deployed to discern numerous emotional states in people.

Gannouni et al. [15] carried out a detailed study to improve emotion recognition’s performance by applying a novel adaptive channel selection method using brain signal activity. This helped to differentiate emotion from one person to another and their emotional states. In order to perform this method, the authors used a DEAP dataset. By using a quadratic discriminant classifier (QDC) and recurrent neural network (RNN), classification and feature extraction are executed for better accuracy rate and emotion classification.

The remainder of this paper is organized as follows: Section 2 describes materials and methods. Section 3 presents the Results. Section 4 presents the Discussion and finally Section 5 presents the conclusion.

## 2. Materials and Methods

### 2.1. Sampling and Data Collection

Data collection was carried out between September 2018 and February 2019 [10]. This experiment was conducted with 750 individuals. Out of this group, 568 young adolescents with age range from 15 to 25 were used from two Lebanese government-aided private schools. Later, from these 568 participants, various selection procedures were followed along with different criteria filtrations and limitations from the school, parents, and individual consent for classification of the emotional state of 22 individuals.

### 2.2. Questionnaire

Questionnaires were in Arabic which is the native language of Lebanon. Time taken to complete the questionnaire is 20 min. Apart from this data collection, we gathered different scales, namely the Toronto Alexithymia Scale [16], Buss and Perry Scale [17], and the Adolescent Depression Rate Scale [18]. These scales evaluate the participants and identify whether they are alexithymic or non-alexithymic adolescents.

### 2.3. Statistical Analysis

For data analysis, we uses Jeffrey’s Amazing Statistics Program (JASP) version 0.16.1 [19]. We performed both general and descriptive analysis. For general analysis, we used age and gender as factors. For descriptive analysis, we used mean ± standard deviation for continuous variables and frequency and percentage for categorical variables. Chi-square tests were used to compare categorical variables. Normal distribution was determined on these main variables, and multivariate analysis of covariance (MANCOVA) was performed to compare multiple measures such as the Toronto Scale and Aggressive and Depression score value at *p* < 0.05.

### 2.4. EEG and EEG Data

The electroencephalogram (EEG) is an electrical movement or signal analyzer where the recording is taken from the brain through the scalp. The recorded waveform shows the cortical electrical activity. It consists of signal intensity and signal frequency. Signal frequency of human EEG waves is classified into delta, theta, alpha, and beta. Each frequency has its own band range such as delta (0–3 Hz). The following Table 1 shows the bands with intervals and their functions. 

Using the EEGLAB toolbox on GNU Octave 6.1, we completed the entire experimental analysis of EEG data. The preprocessing pipeline with sample signals at 250 Hz initially using independent component analysis (ICA) techniques corrected muscle and eye connection errors to eliminate manufactured body movements. The participants who were willing to take part signed the consent form and also a few forms in a formatted procedure. The work was divided into two phases where in first phase we collected the data of alexithymic participants through different scales of measures to find the adolescents who were affected.

For the 568 entrants, the mean age was 18 ± 2.2 years and 302 (53.2%) were females. From the above 568 entrants, we gathered the data of 182 (32.0%) with no alexithymia, 200 (35.2%) with some alexithymia symptoms, and 186 affected by alexithymia. This gave a percentage of 32.74%. We also used two emotional categories, depression and aggression, and calculated the symptoms using visual binning in JASP software to extract the value. The alexithymic adolescents were given preference and those who were willing to consent were taken for further physiological investigation. Thus, there were 22 participants in total.

### 2.5. Methodology

**Figure 2:**
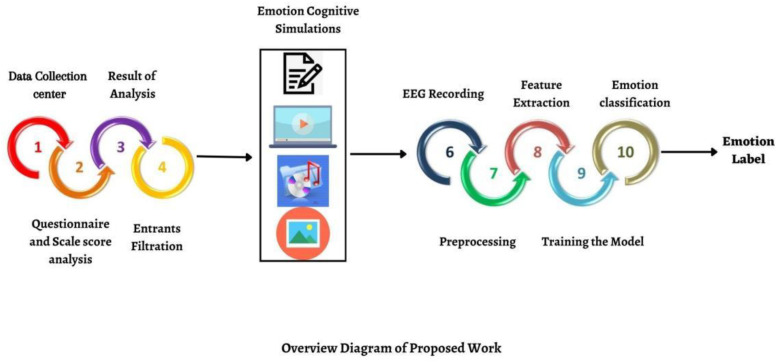
Block diagram of the work.

The process of identifying the complex emotions involved four multimodal emotion cognition stimulations of text, audio, video, and pictorial stimulation. Furthermore, emotion processing using machine learning techniques is elaborately explained in the overview of the work shown below in Figure 2.

We enlisted 22 alexithymic adolescents including 12 males and 10 females. After a general screening process, 7 males and 5 females declined the experiment so there were 7 males and 7 females. The mean age of the 14 participants was 21 years, ranging from 15 to 25. All participants seemed to be very normal in outward appearance. We gathered the Lebanese adolescent data and the range of age was subject to consent of the data owner.

### 2.6. Evaluation

**Figure 3:**
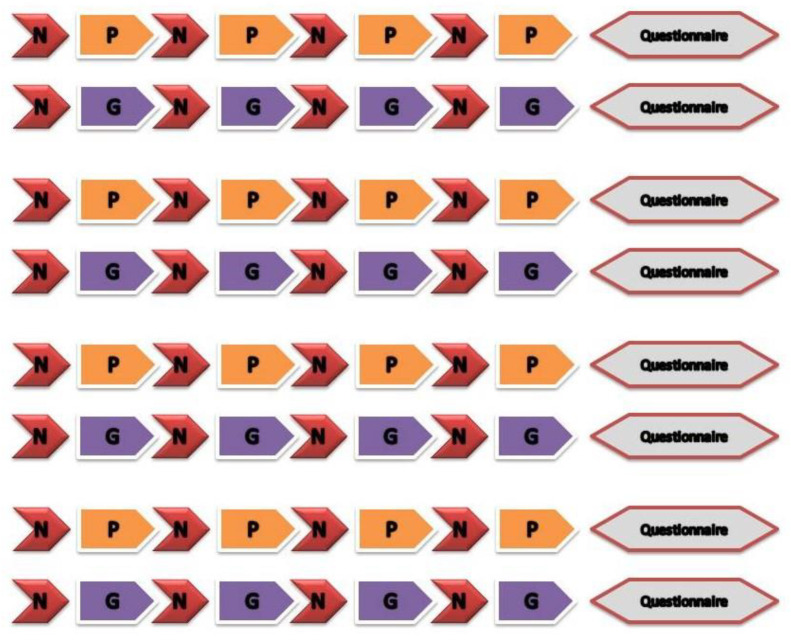
Eight emotional blocks (4 for pleasure emotion and 4 for grief emotion) with questionnaire.

The experiment strategy consisted of an emotional block (four for pleasure draggle and four for grief draggle with interval neural draggle). Each emotional block was divided into a series of 12 s each with a neural draggle of 12 s so the time taken was 84 s. All through the experiment, the EEG signal was recorded in all blocks, as explained in Figure 3. The participants were comfortably seated in an armchair with a screen at a distance of 75 cm and participants were given noise-canceling headphones. Visual representations were set accordingly. The four types of emotion cognition stimulations were text, audio, video, and pictorial stimulation.

**Figure 4:**
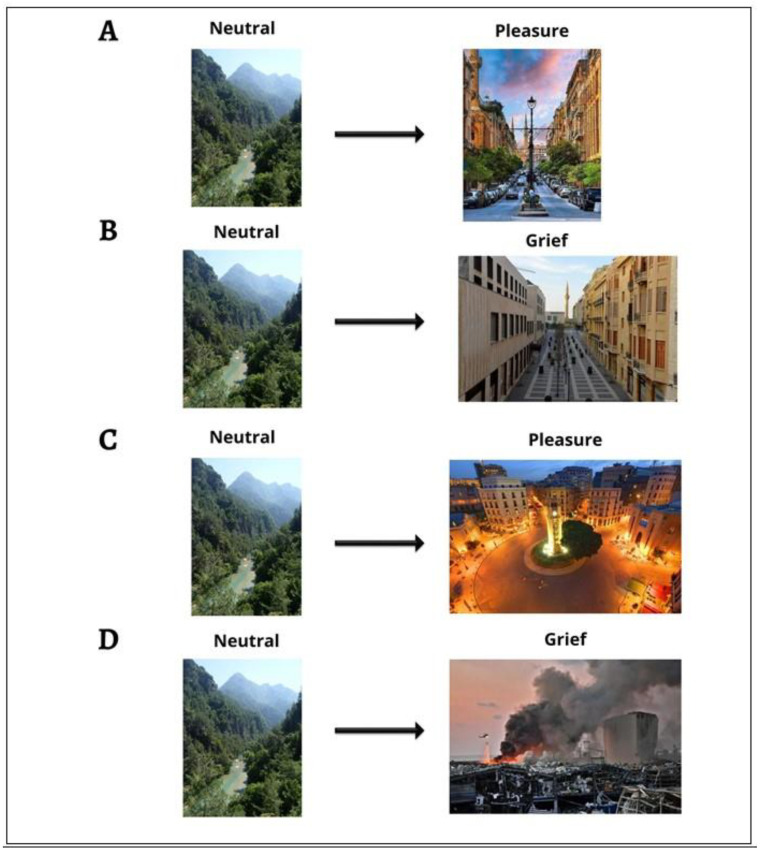
Pictorial stimulation was carried out with two alterative sets in the experiment with (**A**,**C**) for pleasure draggle and (**B**,**D**) for grief draggle.

1. In pictorial stimulations, we used natural scenery pictures representing a neutral scene and other pictures related to inducing emotions of two different categories (pleasure and grief) are shown with the various color schemes accordingly in Figure 4.

2. In audio stimuli, participants heard eight different pieces of music related to emotional induction for 48 s (i.e., 4 for pleasure and 4 for grief). For every pleasure draggle, very pleasant native music was played in the headphones which helped the participants to recall their good memories. Apart from this, we used some manipulated war and military sounds which are unpleasant stimuli for the next interval of time to elicit disgust and portray the unstable nature of Lebanon. We also used neural conditions with a faded colored scene with no sound in between the two emotional stimuli in audio sessions.
3. In video stimuli, entrants were comfortably placed in front of a screen where stimulation video files were played with different emotional stimulations. Apart from these videos, we played some neural videos with a light-colored scheme with low music in between the emotional stimulations in video sessions.
4. With text stimuli, participants were intended to read some self-emotion stimulus text in four intervals of time. Texts were categorized into three statements: pleasing statement, grief statement, and neural statement, as tabulated in Table 2. An EEG cap was placed in all four stimuli sessions to record the spike variation neurons in the brain.

**Table 2:** Samples of statements for text-based stimulation according to the experimental conditions.

Pleasing Statement	Grief Statement	Neutral Statement
He rescued me because he delighted in me	We can’t regain our strength	Homes are built by bricks
Like an apple tree, among the trees in the forest, is my love among you	He doesn’t have to commute to work	Parrots are green
Discipline your son, he will bring delight to you	We can satisfy their need	Beqaa valley is home to Lebanese’s famous vineyards, and wineries
Love always perseveres	Hereafter, we want to be able to cook food in our homes	Lebanon Archs at the rivers of Anjar is good.

In this novel work, we carried out two feature extraction analyses using time frequency analysis and amplitude time series correlations. For analyzing EEG signals, there are three approaches: time domain analysis, frequency domain analysis, time–frequency domain analysis [24]. Time frequency analysis is a technique for recognizing and analyzing brain signals (i.e., EEG signals) using statistics over a wide range of time. The time frequency domain uses complex Morlet wavelet convolution to preserve the knowledge of the temporal dynamics while using EEG data. A complex exponential multiplied by a Gaussian in a complex Morlet wavelet window is demonstrated in the equation [25] below.

(1) $$\mathrm{CMW}=e^{-t^{2}/2s^{2}}\;e^{-i2\pi ft},$$

where

$e^{-t^{2}/2s^{2}}$ is the real valued Gaussian;

$e^{-i2\pi ft}$ is Euler’s formula combined with the sine wave result;

*t* is defined as time centered with regard to the wavelet by taking −2;

*s* is defined as a parameter, expressed as *s* = C2πf;

*C* is the cycle of the wavelet which depends on frequency *f*.

From electrophysiological signals, the 63 × 63 channel matrix’s Spearman correlation was calculated. To eliminate superfluous delta and decrease array size, the highest order upper triangle correlation matrices were combined. The output of the high-dimensionality feature array included 2912 with no windows as the sample for both emotional classes and 12,672 features (pairwise channel correlation coefficient for frequency band). The hardware and software with their package information are displayed in Table 3 below.

### 2.7. Experimental Analysis

**Figure 5:**
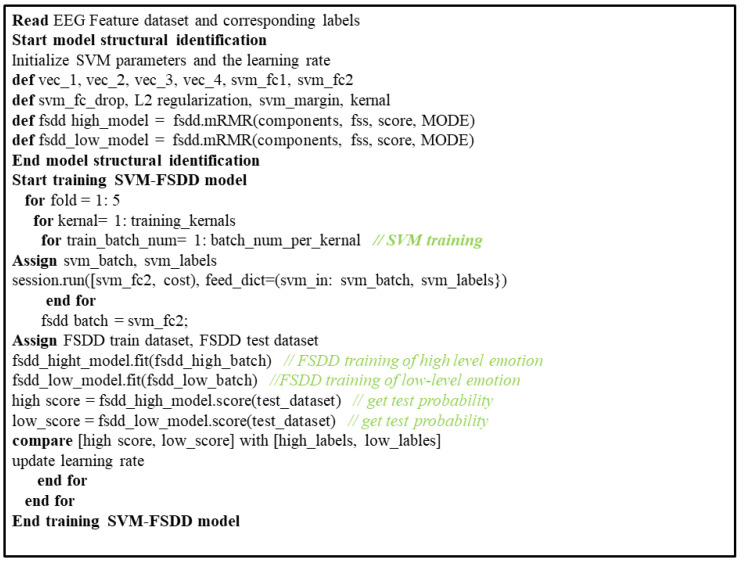
Pseudo code of the detailed procedures for EEG emotion recognition.

The statistics and machine learning toolbox in GNU Octave 6.1 was used for both data visualization and data categorization. We analyzed feature arrays using the support vector machine (SVM) approach for high-dimensional data. We employed a cross-validation technique that employs fivefold cross validation to train and validate the classifier. Each trail’s entire window will move along the same fold. The four draggles (4 pleasure, 4 grief) were taken into consideration for the test draggle with the remaining 40 draggles taken as classifiers. We use 5-fold cross-validation 10 times and averaging across classifiers was performed. We used uniform manifold approximation and projection (commonly known as UMAP) [32]. It is a dimension reduction technique used for data visualization of the dataset. It is similar to t-SNE. The data were classified using the UMAP algorithm, which was customized to the feature sets (time frequency/frontal alpha symmetry (FAA) and correlational features) of the participants. They were also examined to see if the data clustering and expression of the participants’ emotions could be distinguished. Due to the enormous dimensionality of the dataset utilized in the proposed system, we implemented feature selection using the feature ranking technique in GNU Octave. The maximum relevance minimum redundancy method (mRMR) [33] is a feature extraction and selection method and helps to reduce computing complexity and create models with the best generalization, and it tends to improve the classifier’s learning efficiency [34]. In binary classification using τ-tests for independent precedents, feature selection plays a vital role between the classes. In order to improve the test, we differentiated the emotional state using a smaller set of features and 5-fold cross-validation. The pseudo code for the step-by-step method of emotion classification is shown in Figure 5 below.

## 3. Results

There are three main results from the proposed system.

1. To determine whether the two distinct emotional states can be rigorously separated in a categorized way using time frequency/FAA and correlational features retrieved within 1 s windows, a machine learning approach is investigated.
2. Identifying two complex emotions such as pleasure and grief through common highest ranked features through feature selection distance discriminant (FSDD) where necessary spectral and spatial properties are identified.
3. The subjective emotion experienced is explored by self-evaluation (i.e., a questionnaire) after each multimodal visual aid where it excludes the subject-dependent classification.

### 3.1. Classification of Complex Emotion Using SVM

**Figure 6:**
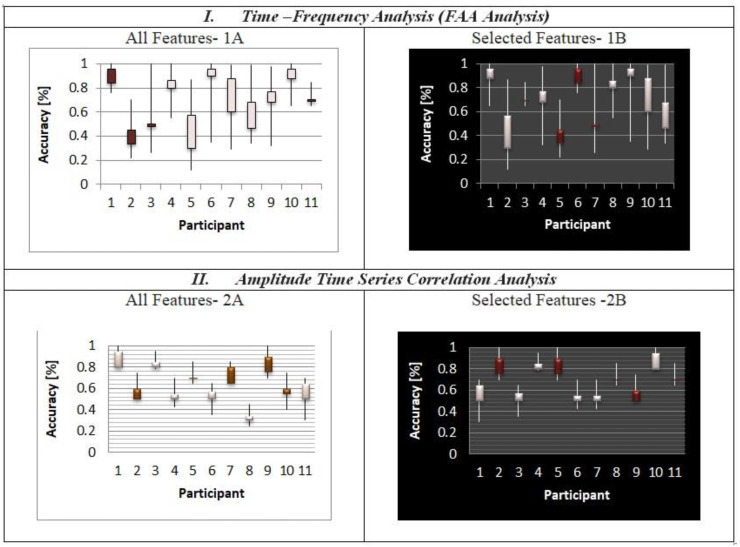
The outcome of classification using each feature extraction method’s 100 chosen features (**1B**,**2B**) and all available features (**1A**,**2A**), respectively.

For each participant, the process of cross-validation was performed and all the results are shown in Figure 6. For all the features in time frequency/FAA analysis after the cross-validation, the accuracy ranged from 60.22% up to 93.1% (where mean = 80.02, SD = 2.92) as shown in Figure 6-1A. In amplitude time series correlation analysis, the accuracy ranged from 63.4% to 79.2% (where mean = 71.23, SD = 2.89) as shown in Figure 6-2A. This work focuses on the considerable intersubjective variability seen in both feature sets. The feature selection distance discriminant was used next. With a chosen subset of 100 features, we applied a 5-fold cross-validation approach for this. The chosen feature significantly increased the system’s capacity to predict the time frequency/FAA feature (*p* = 0.024). In Figure 6-1B, the accuracy obtained from 100 selected features using the feature selection distance discriminant (FSDD) algorithm and 5-fold cross-validation ranges from 62.9% to 94.1% (where mean = 82.02, SD = 2.89). In contrast to performance, significance (*p* ≤ 0.0001) of the amplitude time series correlation analysis is given in Figure 6-2B with accuracy ranging from 64.2% to 82.2% (mean = 78.38, SD = 3.64), which is in contrast to the prior results of all characteristics. As a result, both subsets of the chosen feature highly recommend the process and system’s performance (*p* ≤ 0.0001).

**Figure 7:**
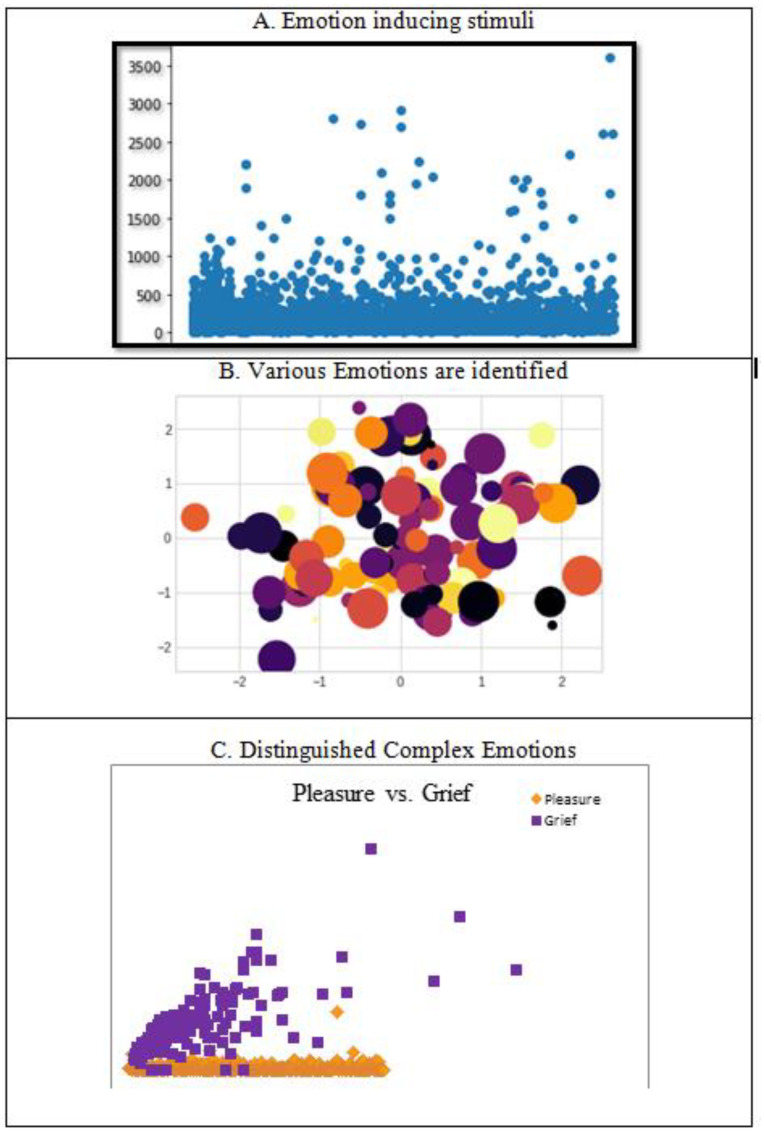
The emotions induced by the stimulus to which participants were exposed have three different classifications of complex emotions.

For better understanding of the two feature classes of pleasure and grief, we used the data visualization algorithm to classify the two different classes using UMAP. The performance of the subjective dataset of Participants 1 to 5 whose best and worst classification accuracies were identified in the cluster of emotions from global geometry of participants was divided between two emotional states. From the EEG signals, we extracted standard emotions such as happy, angry, and sad as shown in Figure 7A. In contrast to the global geometry for Participants 1 to 5, we classified emotions with different colors in Figure 7B. For amplitude time series correlation analysis, the distinguished emotions (pleasure and grief) were extracted with minimal complexity, as the emotions are complex, to distinguish them from other common emotions as indicated with pleasure in orange and grief in purple in Figure 7C. This gives a more refined regional classification than sample clustering into lines.

### 3.2. Feature Selection across Participants

**Figure 8:**
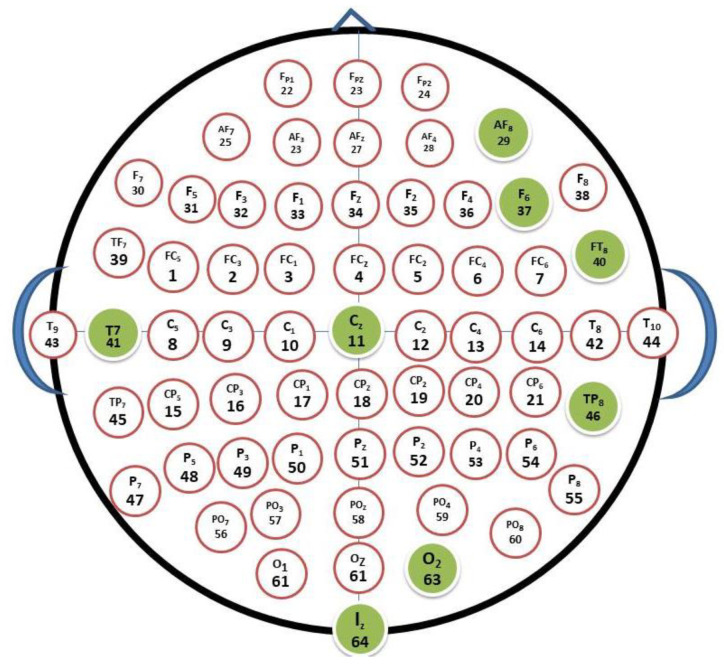
Highly ranked features of classification are extracted from the time–frequency analysis. Highlighted channel plots are engaged in identifying the complex emotions with 280 features in total.

The top 20 features are extracted for each of the 14 entrants. The discrimination between the two complex emotions is the topic of these abridged aspects. Figure 8 illustrates the significant 280 time frequency/FAA analysis elements. For this, we did not use the FAA coefficient; instead, we generalized the feature that was taken from the section and hemisphere of the brain. When compared to the right hemisphere at a ratio of 4:1 (45:14 features), the left hemisphere has a very significant relevance in identifying things on the frontal side. The left hemisphere was evaluated using channels set up with set pairs such as FP1/FP2, AF3/AF4, AF7/AF8, F1/F2, F3/F4, F5/F6, and F7/F8.

**Figure 9:**
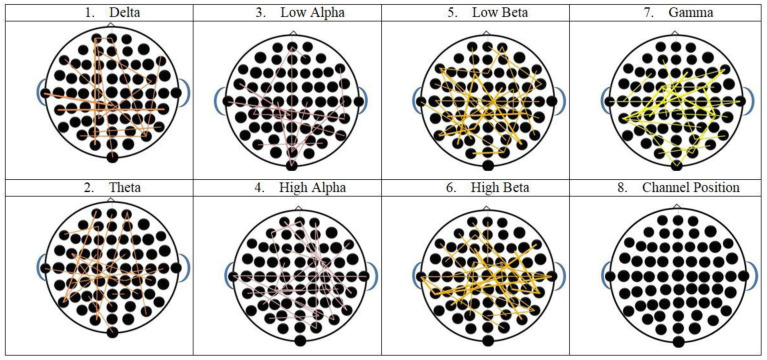
Identification of the complex emotions with the most significant qualities, amplitude time series correlation analysis was carried out.

These channels were chosen from the EEG cap as the most important ones to extract the characteristics from. Figure 8 illustrates the channel’s structured setup. We selected a few key channels from these channels to display. When compared to complementary right hemisphere channel AF8, AF4 pertains to the left hemisphere. Gamma and beta were thus designated as the main bands in terms of characteristics. Both hemispheres include the temporal regions O1, O2, and O3. Figure 9 shows the seven frequency bands used in the calculations of the amplitude time correlation analysis. The theta and high alpha bands, in particular, show an uneven correlation pattern.

The ratio of features involved in this observation is around 2:1 (46 to 20 features). The frontal side is included by the channels FP1/FP2, AF3/AF1, AF7/AF8, F1/F2, F3/F3, F5/F6, and F7/F8. The correlation of these left and right frontal channels across all frequency bands was completed. In Table 4, we see that AF7 is highly linked with the left hemisphere channel and very alpha in the right hemisphere.

### 3.3. Feedback Rating and Correlation with SVM Performance

Feedback reviews based on multimodal stimulation (text, audio, video) are highly impacted in the system. We calculated the emotional intensity, and the use of text-based stimulation and concentration level were closely observed and reported. Emotional intensity was calculated from “mild to high” for the initial and last blocks with an increase in the second and fourth blocks. Correlation analysis between feedback rating and classifier performance with both feature sets was not significant for any measure (*p* > 0.05).

## 4. Discussion

**Figure 10:**
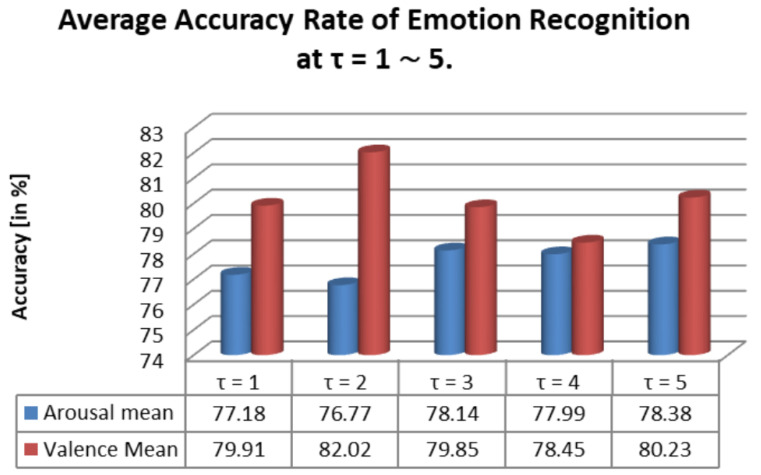
Calculation of average accuracy of emotion recognition with τ-values of other studies.

In our proposed system, we demonstrated the potential for using ML and EEG algorithms to identify multidimensional states. We identified a multimodal stimulus, such as an audio and video stimulus, that could be used in an affective computing experiment. That is what we demonstrated: EEG is a reliable electrical signal tool for differentiating diverse discrete emotions such as pleasure and grief, despite low complex states and the inability to infer action in a brain region. The was carried out by utilizing correlational features computed with a 1 s window FSDD and time-frequency/FAA. Furthermore, we were concerned with separating the most elements for categorization across participants. To determine this, we used a feature selection approach and calculated continuous cross-validation using the 100 sub features that were chosen. Based on the study’s findings, we demonstrate FSDD feature extraction with channels from the frontal left hemisphere and the occipital lobe. When testing the model with all features utilizing time–frequency and FAA features, we demonstrate accuracy ranging from 60.22% to 93.1%. Figure 10 depicts a practical model performance with a feature chosen for its minimal accuracy (63.4%) and maximum accuracy (79.2%) from the amplitude time series correlation analysis.

Several studies have found that each person processes emotions in their own unique way [35,36,37,38], emphasizing the importance of individual data analysis. When focusing on the literature, there are a few drawbacks that lead us to conclude that we should not use any antique expulsion or epoch demission in ICA to remove muscular and eye blink artefacts. We focused on our proposed approach’s robust classification performance in a real-world scenario. When looking at the highest-ranked features across participants, the significance of FAA elements, as discovered in previous characterization reviews including pleasure [3], was not revealed. This work is compared to various fundamental emotion categorization algorithms, and the proposed system outperforms other algorithms in terms of accuracy percentage, such as LSTM, HMM, ANN, and CapsNet. The comparison is tabulated in Table 5.

## 5. Conclusions

This study uses multimodal data stimulation to generate emotion in adolescent Lebanese citizens in order to categorize complicated emotions, making it appear to be a realistic goal. Two complex emotions, pleasure and grief, were demonstrated in a lab setting. We suggest a technique that uses real-time data processing to elicit an affective state based on an EEG. As online setting features are identified using temporal and spatial parts with a majority of the left frontal site, which discriminates the two complex emotions easily, this paper demonstrates that SVM classification exhibits the discrete emotion using EEG signals in different contraceptive manners, including short time windows. Additionally, left frontal and right frontal features specifically extracted from AF7’s EEG channel were used and compared to AF8. The human brain’s frequency band suggests that the channels are more beneficial in recognizing and separating two emotions. High-frequency bands of beta and gamma are involved. From all of them, the EEG-based results provide a valuable tool for identifying and classifying discrete emotions that is applied with real-time BCI that is EEG-based.

## Figures and Tables

**Table 1:** EEG signals with their band intervals.

Band Name	Band Intervals (Hz)	Functions
Delta	<3	Related to the unconscious mind and it transpires in deep sleep
Theta [20]	4–7	Related to the subconscious mind and transpires in sleeping and dreaming
Alpha [21]	8–15	Related to a relaxed mental state associated with brain activation
Beta [22]	16–31	Related to active mind state and transpires during highly engrossed mental activity
Gamma [23]	>31	Related to hyperfocused brain activity

**Table 3:** Hardware and software specification.

Sl. No	Name	Version	Description
1	Operating System: Ubuntu [26]	16.04	Linux operating system
	Intel i7 Processor	8th Gen	
	8 GB DDR4 RAM		
2	Anaconda [27]	2019.03	Python framework for scientific computing and large-scale data processing, programming language
3	Python [28] Pyspark [29]	3.9 2.4.4	Interface for Apache Spark to analyze the data in distribution environment
4	GNU Octave [30]	6.1	High-level language framework mainly used for numerical computation by solving linear and non-linear problems numerically compatible even with MATLAB
5	EEG Lab [31]	9.0.7.6	GUI to interact with the high-density EEG and other interactive toolboxes for processing continuous data and data related to EEG, MEG, and brain electrophysiological signals
Python Libraries and Packages			
Conda install, pip install, Matplotlib, numpy, pandas, pytorch, SkylearnV 0.21			

**Table 4:** EEG channel labeling and channel points in brain areas.

Sl. No	Brain Area	Left Hemisphere	Total Features	Right Hemisphere	Total Features
1	Frontal	Fp1, AF3, AF7, F1, F3, F5, F7	35	FP2, AF4, AF8, F2, F4, F6, F8	11
2	Temporal	FT7, FT9, T7, TP7, TP9	32	FT8, FT10, T8, TP8, TP10	36
3	Central–Pariental	FC1, FC3, FC5, C1, C3, C5, CP1, CP3, CP5, P1, P3, P5, P7	39	FC2, FC4, FC6, C2, C4, C6, CP2, CP4, CP6, P2, P4, P6, P8	20
4	Occipital	PO3, PO7, O7	19	PO4, PO8, O2	15
5	Midline	FPz, Fz, Cz, CPz, POz, Oz			15

**Table 5:** Comparative analysis of current work with other existing related studies.

Study Authors	Algorithms	Features	Evaluation Mode	Accuracy (%)	
				Arousal	Valence
Chao et al. [39]	CapsNet	Multiband feature matrix	10-fold cross-validation	68.28	66.73
Xing et al. [40]	LSTM	Frequency band power	10-fold cross-validation	74.38	81.10
Chen et al. [41]	HMM	Fusion feature	5-fold cross-validation	73.00	75.63
Mert and Akan [42]	ANN	MEMD-based feature	Leave-one-trail-out validation	69.10	71.99
Proposed Method	SVM	Feature selection distance discriminant (FSDD)	5-fold cross-validation	80.20 ± 2.92	82.02 ± 3.64

## Data Availability

Not applicable.
